# Global trends and research status in ankylosing spondylitis clinical trials: a bibliometric analysis of the last 20 years

**DOI:** 10.3389/fimmu.2023.1328439

**Published:** 2024-01-08

**Authors:** Wenhui Zhang, Meng Li, Xuhao Li, Xingxin Wang, Yuanxiang Liu, Jiguo Yang

**Affiliations:** ^1^ College of Acupuncture and Massage, Shandong University of Traditional Chinese Medicine, Jinan, Shandong, China; ^2^ Department of Neurology, Affiliated Hospital of Shandong University of Traditional Chinese Medicine, Jinan, Shandong, China

**Keywords:** ankylosing spondylitis, clinical trials, bibliometric analysis, placebo-controlled trials, Citespace, VOSviewer, interleukin-17

## Abstract

**Background:**

Ankylosing spondylitis (AS) is a rheumatic and autoimmune disease associated with a chronic inflammatory response, mainly characterized by pain, stiffness, or limited mobility of the spine and sacroiliac joints. Severe symptoms can lead to joint deformity, destruction, and even lifelong disability, causing a serious burden on families and society as a whole. A large number of clinical studies have been published on AS over the past 20 years. This study aimed to summarize the current research status and global trends relating to AS clinical trials through a bibliometric analysis.

**Methods:**

The Web of Science Core Collection database was searched for publications related to AS clinical trials published between January 2003 and June 2023. Bibliometric analysis and web visualization were performed using CiteSpace, VOSviewer, and a bibliometric online analysis platform (https://bibliometric.com), which included the number of publications, citations, countries, institutions, journals, authors, references, and keywords.

**Results:**

1,212 articles published in 201 journals from 65 countries were included in this study. The number of publications related to AS clinical trials is increasing annually. The United States and the Free University of Berlin, the countries and institutions, respectively, that have published the most articles on AS, have made outstanding contributions to this field. The author with the most published papers and co-citations over the period covered by the study was Desiree Van Der Heijde. The journal with the most published and cited articles was Annals of the Rheumatic Diseases. The keywords: “double-blind,” “rheumatoid arthritis,” “efficacy,” “placebo-controlled trial,” “infliximab,” “etanercept,” “psoriatic arthritis” and “therapy” represent the current research hotspots regarding AS.

**Discussion:**

This is the first study to perform a bibliometric analysis and visualization of AS clinical trial publications, providing a reliable research focus and direction for clinicians. Future studies in the field of AS clinical trials should focus on placebo-controlled trials of targeted therapeutic drugs.

## 1 Introduction

Ankylosing spondylitis (AS), also known as axial spondyloarthritis (1, 2), is an immune-mediated, chronic inflammatory disease that mainly affects the spinal and sacroiliac joints (3). The clinical symptoms include pain, stiffness, and limited movement (4). The cause of the disease is unclear, with clinical studies having found that the prevalence of this disease is approximately two to three times greater in men than it is in women (5), and that it is most heavily related to genetics, infection, and trauma. According to statistics, the heritability of the disease is greater than 90% (1) and is mostly related to HLA-B27 genetic factors. In addition to spinal and peripheral joint involvement, ulcerative colitis, uveitis, and psoriasis have also been shown to occur clinically (6). The disability rate associated with this disease is high, seriously affecting the quality of life of patients and causing significant social and economic burdens. Nonsteroidal anti-inflammatory drugs and tumor necrosis factor inhibitors are commonly used for the clinical treatment of this disease (7–9). Recent studies have focused on interleukin inhibitors as a new type of targeted therapeutic drug (10).

Clinical trials, also commonly referred to as randomized controlled trials (RCT), are currently the most powerful method for evaluating interventions in clinical practice. They are scientific research activities used to determine the effectiveness and safety of a given treatment (11). Clinical trials encompass a rigorous study design and provide solid evidence based on reliable results that can be used to guide clinical treatment practices (12). Since the discovery of the first biological agent for the treatment of AS, many clinical trial articles have been published in this field (13), exploring the single and combined effects of various treatments. This study identifies the next research direction in this area by integrating and analyzing the results of these clinical trials.

Bibliometric analysis is used for the quantitative analysis of research hotspots and global trends in a specific field (14). Analysis software is used to perform bibliometric analysis of the relevant countries, authors, journals, and keywords in specific research fields (15). Although many reviews have been published on the different treatment approaches for AS, there remains a lack of a systematic bibliometric analysis of AS clinical trials. Thus, this study aims to review articles published on AS over the past 20 years to help clinicians gain a better understanding of the relevant research hotspots and frontiers in this field.

## 2 Materials and methods

### 2.1 Data sources and search strategies

**Figure 1:**
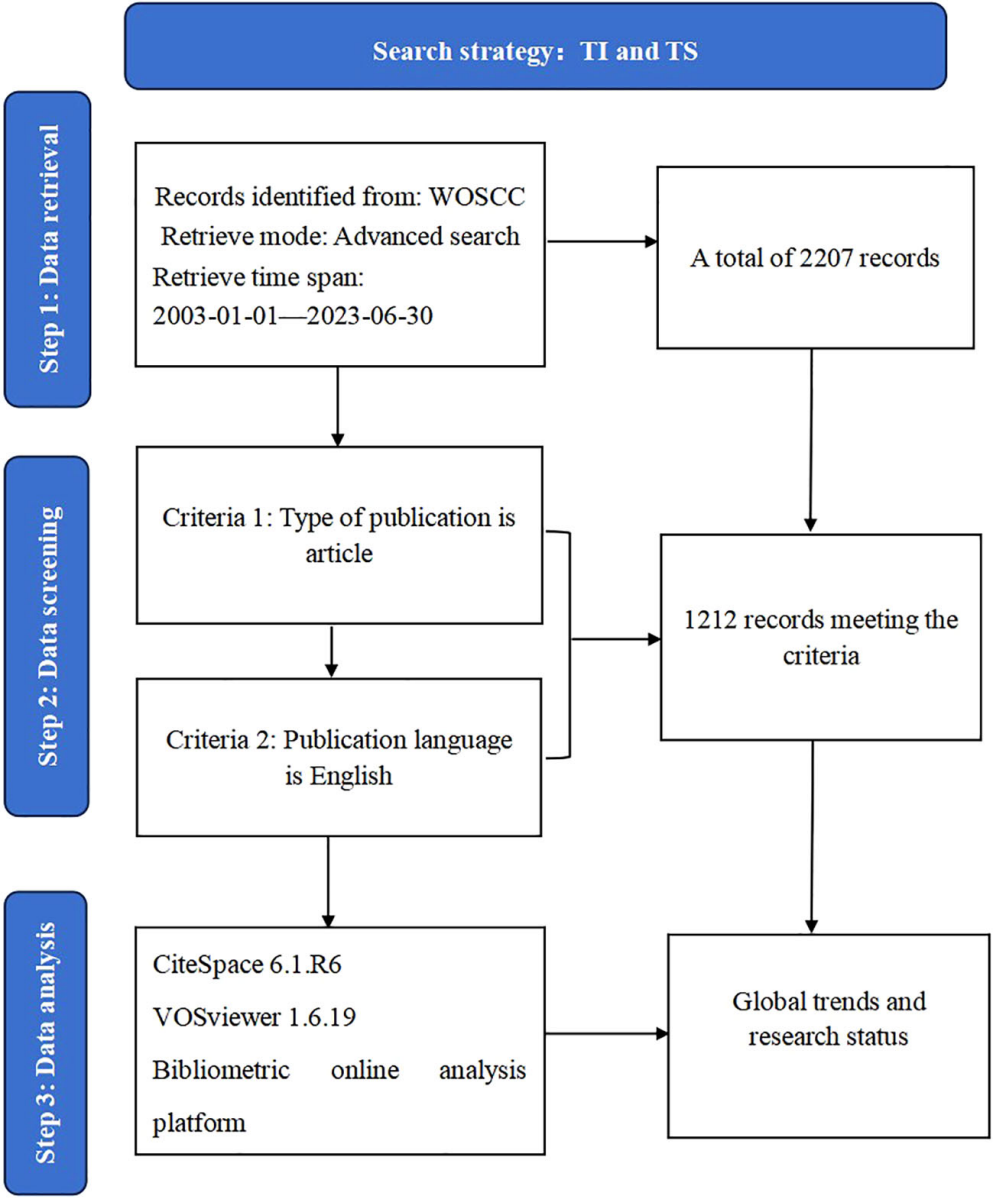
Flow chart of literature retrieval in research.

The articles in this study were indexed from the Web of Science Core Collection (WoSCC) database, which contains more than 10,000 journals and is one of the most commonly used and authoritative databases for bibliometric analysis (16). The search strategy used was as follows (17): TI = (“Spondyloarthritis Ankylopoetica*”) OR TI = (“Ankylosing Spondylitis*”) OR TI = (“Ankylosing Spondylarthritis*”) OR TI= (“Spondylarthritis Ankylopoetica*”) OR TI= (“Spondyloarthritides, Ankylosing*”) OR TI= (“Ankylosing Spondyloarthritis*”) OR TI= (“Ankylosing Spondyloarthritides*”) OR TI= (“Spondylitis Ankylopoetica*”). AND TS= (“Clinical Trial”OR “Controlled Clinical Trial”OR “Randomized Controlled Trial”). The search period covered was from January 1, 2003, to June 30, 2023. The document type was simultaneously limited to “article,” and our search was limited to articles published in English. A flowchart describing how publications were selected is presented in **Figure 1**.

### 2.2 Data extraction and analysis

CiteSpace(6.2.R4) is a visualization software developed by Dr. Chaomei Chen of Drexel University in Philadelphia, Pennsylvania, USA, which analyzes the strongest citation bursts of references and keywords to identify relevant hotspots and trends in a given research field. VOSviewer(v1.6.19) is a quantitative analysis software developed by Nees Jan van Eck and Ludo Waltman at Leiden University in the Netherlands that constructs visual network maps. This study used VOSviewer to analyze collaborative networks between countries, institutions, journals, and authors, identify reference co-citations, and map keyword overlay visualization. Each node represented a specific parameter; the larger the node, the heavier the parameter. Different clusters have differently colored nodes and lines. The thicker the connection line between the nodes, the stronger the link. The distribution of cooperation between countries was analyzed using the online analysis platform at https://bibliometric.com.

### 2.3 Research ethics

Ethical approval was not required because no patients or animals were included in this study.

## 3 Results

### 3.1 Annual growth trends in publications

**Figure 2:**
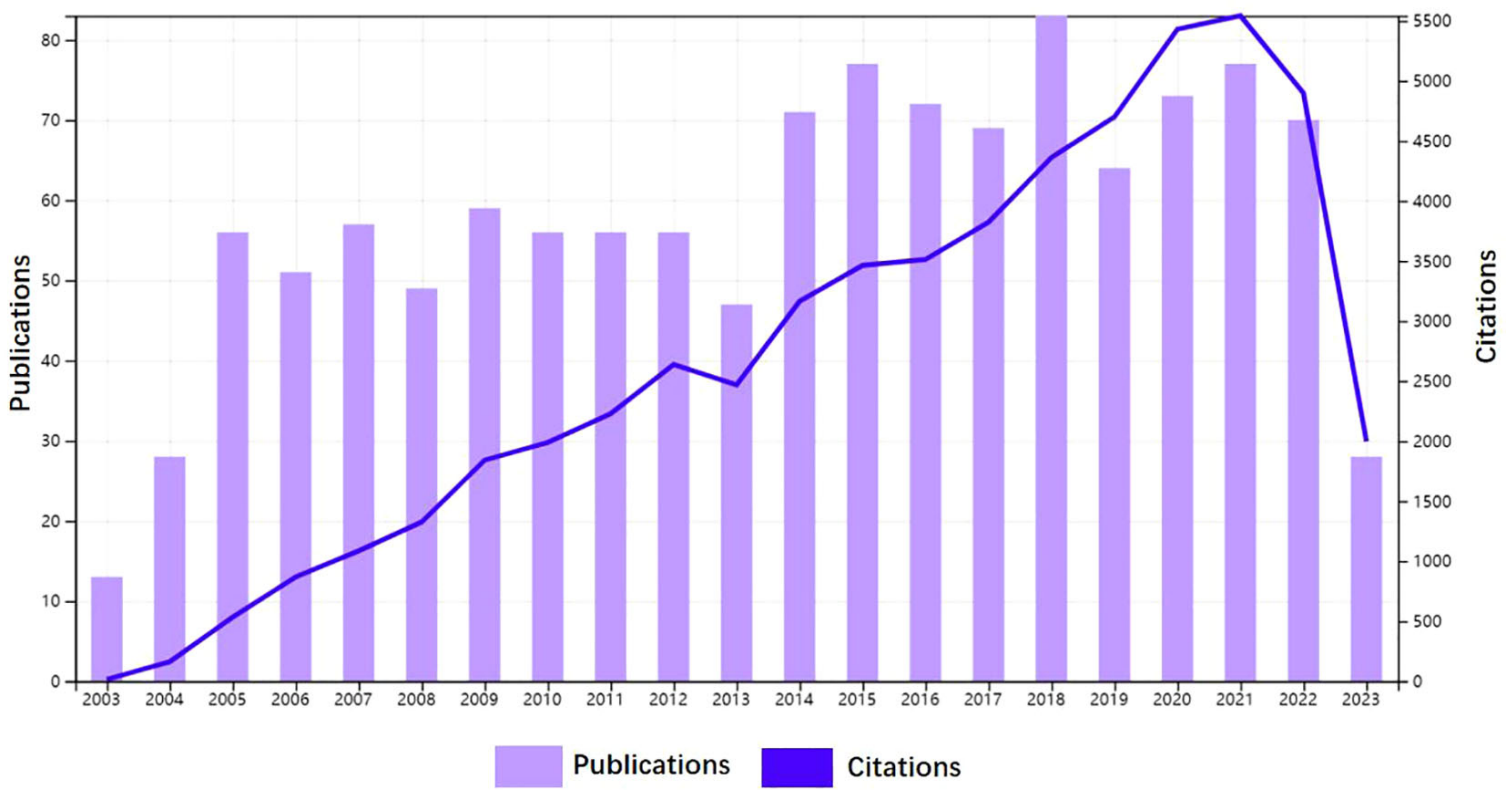
The annual publications and citations of AS Clinical trial articles from 2003 to 2023.

From January 1, 2003, to June 30, 2023, 1,212 clinical trial publications on AS were retrieved from the WOSCC database. **Figure 2** shows the number of clinical trial publications published on AS in the past 20 years. Although there were slight fluctuations between 2013 and 2019, the overall trend is of a steady increase, indicating that clinical trial research on AS has received increasing attention from researchers. The number of publications peaked in 2018 with 83 articles published and 4,371 citations made. The publications included in this study received 56,425 citations in total, with an average citation frequency of 46.33. Between 2003 and 2023, the H-index for academic fields is 114.

### 3.2 Distribution of countries/regions and institutions

**Figure 3:**
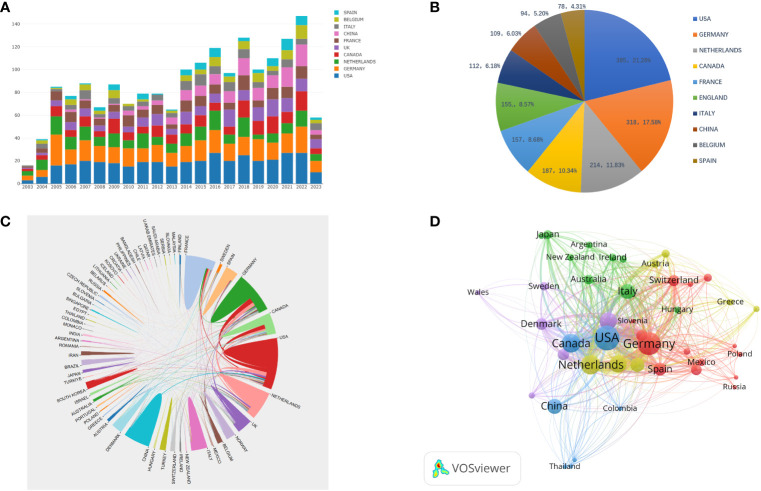
**(A)** Annual publishing trends in the top 10 countries from 2003 to 2023; **(B)** The proportion of the 10 most productive countries in the field of AS clinical trials over the past 20 years; **(C)** A visual map of international cooperation in countries and regions; **(D)** Network visualization showing relationships between countries.

**Figure 4:**
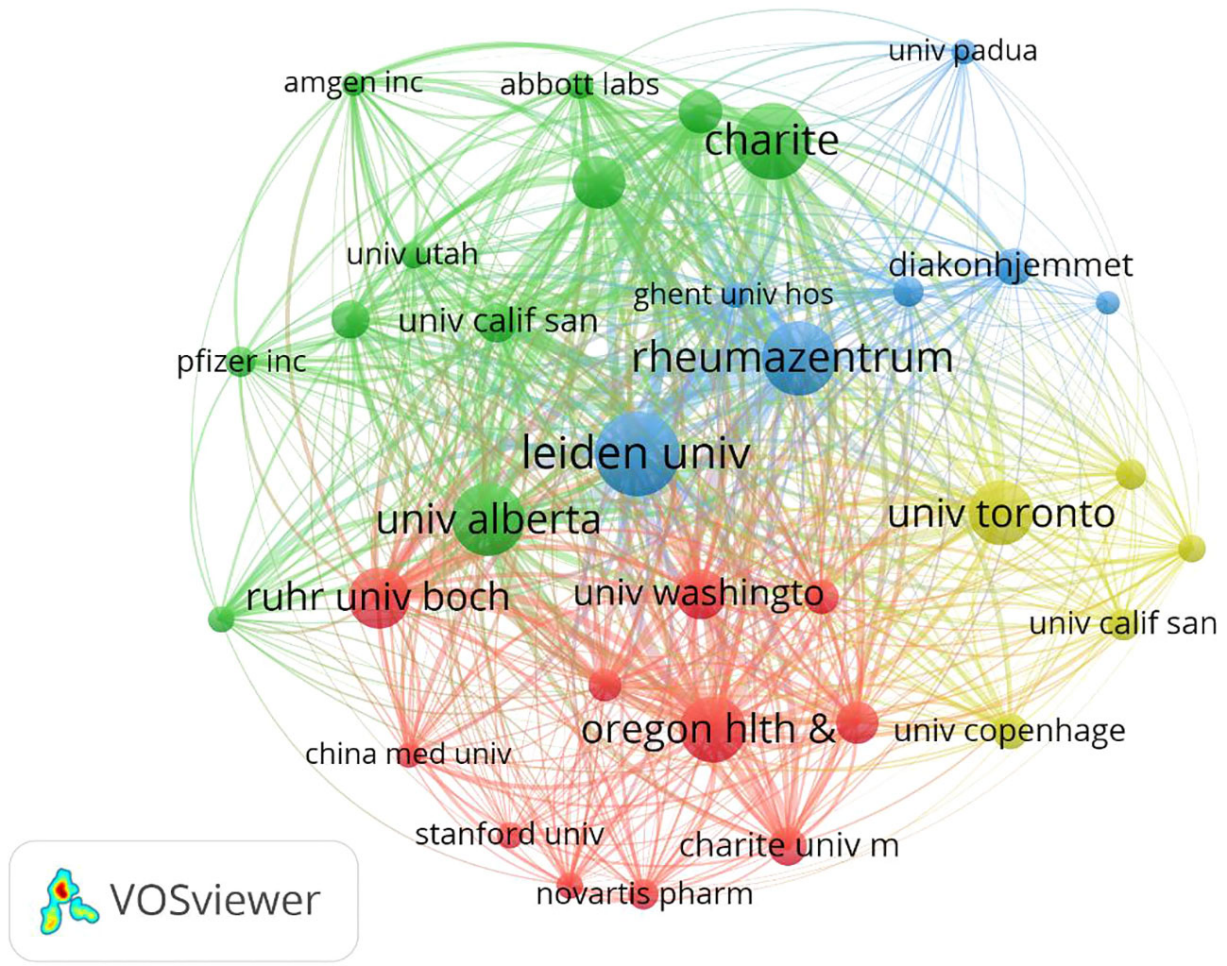
Web visualization of institutional citation analysis.

**Table 1** shows the top 10 most prolific countries and institutions in the field of AS clinical trials, as well as their total number of publications (NP), total citations (NC), average citations (AC), and H-index scores. Among these countries, the United States had the most published papers (NP= 385, 21.28%), followed by Germany (NP=318, 17.58%). The country with the highest number of citations was Germany (NC=27,066), followed by the United States (NC=24,454) and the Netherlands (NC= 19,034). The country with the highest average number of citations was the Netherlands (AC=88.94), followed by France (AC=85.31) and Germany (AC=85.11). It is worth noting that among the 10 most productive countries, the United States had a significantly higher NP than Germany and the Netherlands, but a relatively low AC. By comparison, China’s NP is approximately the same as that of Italy and Belgium, while China’s NC and AC are both relatively low. **Figure 3A** shows the number of annual publications in the top 10 most productive countries, demonstrating a steady increase in the number of publications relating to AS clinical trials in each country. **Figure 3B** shows the proportion of publications in the 10 most productive countries in the past 20 years. Together, the three most productive countries the United States, Germany, and the Netherlands account for more than half of the publications in this area. **Figure 3C** represents a map of international cooperation between countries, and shows that the United States works most closely with Germany and Canada. Citation relationships between countries were analyzed using VOSviewer (**Figure 3D**). Only countries with at least five publications were included in the analysis. Of the 43 countries and territories that reached this threshold, the top five based on Total Link Strength (TLS) rankings were The United States (TLS=937), Germany (TLS=862), the Netherlands (TLS=646), Canada (TLS=566), and the United Kingdom (TLS=483). **Table 1** summarizes the 10 most influential institutions in this area. The institution with the highest NP value was the Free University of Berlin (NP=184), followed by Charite Universitatsmedizin Berlin (NP=179) and Humboldt University of Berlin (NP=179). Of the top 10 institutions, the top five were all from Germany, followed by three from France and two from the Netherlands. VOSviewer was used to analyze the citation relationships between institutions (**Figure 4**). Only the institutions with at least 20 publications were included in the analysis. Of the 34 that reached this threshold, the top five with the highest TLS rankings were Rheumazentrum Ruhrgebiet (TLS=4043), Leiden University (TLS=3992), Charite Universitatsmedizin Berlin (TLS=3194), the University of Alberta (TLS=3046), and Oregon Health Science University (TLS=2603).

**Table 1:** Top 10 countries or regions and institutions that have contributed to clinical trial research for AS.

Rank	Country	Number of publications	Number of citations	Average Number of citations	H-Index	Institutions	Number of publications	Number of citations	Average Number of citations	Location
1	USA	385	24454	63.35	79	Free University of Berlin	184	17982	97.73	GERMANY
2	GERMANY	318	27066	85.11	89	Charite Universitatsmedizin Berlin	179	17027	95.12	GERMANY
3	NETHERLANDS	214	19034	88.94	66	Humboldt University of Berlin	179	17027	95.12	GERMANY
4	CANADA	187	13074	69.91	58	Rheumazentrum Ruhrgebiet	153	15286	99.91	GERMANY
5	FRANCE	157	13393	85.31	54	Ruhr University Bochum	149	14979	100.53	GERMANY
6	ENGLAND	155	8001	51.62	48	UDICE-French Research Universities	122	10675	87.5	FRANCE
7	ITALY	112	5799	51.78	36	Assistance Publique Hopitaux Paris (APHP)	111	10027	90.33	FRANCE
8	CHINA	109	2147	19.70	23	Universite Paris Cite	111	9991	90.01	FRANCE
9	BELGIUM	94	7454	79.30	39	Leiden University	106	9575	90.33	NETHERLANDS
10	SPAIN	78	3919	50.24	31	Leiden University Medical Center (LUMC)	103	9387	91.14	NETHERLANDS

### 3.3 Authors and co-citation authors

**Figure 5:**
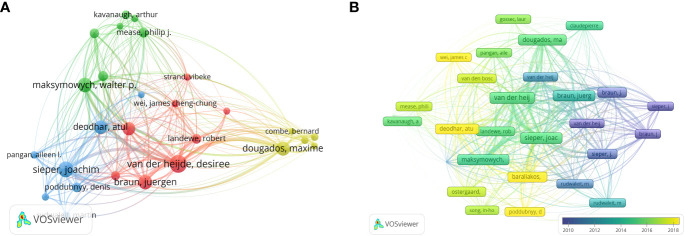
**(A)** Network visualization of co-authored analysis. **(B)** Author citation analysis overlay visualization based on VOSviewer. Purple nodes represent authors involved in earlier studies in the field, while yellow nodes represent authors engaged in later studies.

The analysis of the 1,212 articles included in this analysis showed that 5,448 authors contributed to the publication of the included AS clinical trial articles. **Table 2** shows the top 10 most productive and co-cited authors in the field of AS clinical trials. Desiree Van Der Heijde has been the most productive author, with 126 articles and 15,139 citations, followed by Joachim Sieper (NP=85, NC=9115) and Juergen Braun (NP=80, NC=11276). Van Der Heijde was also the most-cited author, with 627 citations, and ranked second in terms of centrality (0.06). Xenofon Baraliakos was the author with the highest centrality ranking (0.11). The minimum number of author publications was set to 15, and 27 authors were selected for the author co-analysis using VOSviewer. Co-author analysis (**Figure 5A**) grouped the authors into four clusters. The red cluster (eight authors) represents the largest cluster of co-authors. Subsequently, author citation analysis was performed using the VOSviewer software. The overlay visualization (**Figure 5B**) shows that Sieper and Braun are the authors most associated with early stage clinical trial studies. Van Der Heijde is the most influential author in the mid-stage, while Atul Deodhar and Baraliakos are the emerging authors with regard to the clinical trial studies published in recent years. The top three authors based on TLS were Van Der Heijde (TLS = 2,933), Sieper (TLS = 2,835), and Braun (TLS = 2,254).

**Table 2:** The top 10 most productive authors and co-cited authors who contributed to AS clinical trial research.

Rank	Author	Number of publications	Number of citations	Average Number of citations	H-Index	Co-cited Author	Number of citations	Centrality
1	Van Der Heijde, Desiree	126	15139	120.15	61	Van Der Heijde, Desiree	627	0.06
2	Sieper, Joachim	85	9115	107.24	47	Braun, Juergen	590	0.03
3	Braun, Juergen	80	11276	140.95	52	Sieper, Joachim	391	0.04
4	Maksymowych, Walter P	71	6116	86.14	37	Van Der Linden, S	384	0.05
5	Baraliakos, Xenofon	70	5675	81.07	32	Calin, A	383	0.04
6	Landewe,ROBERT B.M	67	8230	122.84	41	Garrett,S	370	0.04
7	Deodhar, Atul	61	3487	57.16	29	Rudwaleit, Martin	347	0.04
8	Dougados, Maxime	43	3789	88.12	24	Dougados, Maxime	344	0.07
9	Rudwaleit, Martin	42	4694	111.76	29	Davis,John C	251	0.04
10	Listing, Joachim	41	5687	138.71	34	Baraliakos, Xenofon	243	0.11

### 3.4 Journals and co-cited journals

**Figure 6:**
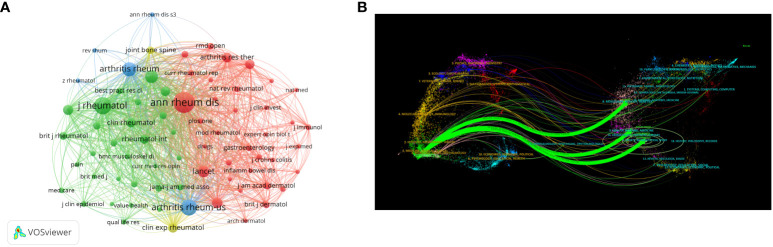
**(A)** Web visualization of journal co-citation analysis. **(B)** The dual map overlay of journals.

A total of 1,212 articles related to clinical trials on AS were published in 251 journals. **Table 3** shows the top 10 journals and top 10 co-citations. The journal with the most published papers was Annals of the Rheumatic Diseases (n=137, 23.50%), which was also the most cited journal (NC=15,592), followed by the Journal of Rheumatology (n=111,10.04%) and Rheumatology (n=64,10.98%). Annals of the Rheumatic Diseases (NC=8,580) was also the most commonly cited journal, followed by the Journal of Rheumatology (NC=4,473). In addition, four of the top ten co-cited journals had an impact factor (IF) greater than 10, with the Lancet Journal having the highest IF, followed by the New England Journal of Medicine. **Figure 6A** shows the co-citation analysis of journals using the VOSviewer software. Only journals with at least 60 published articles were included, and 70 journals were selected for the analysis. The top three biggest journals in terms of TLS were Annals of the Rheumatic Diseases (TLS = 213,896), Journal of Rheumatology (TLS = 124,400), and Arthritis and Rheumatism (TLS = 114,740). The dual map overlay of journals (**Figure 6B**) shows three main citation pathways: Papers published in Health/Nursing/Medicine, Sports/Rehabilitation/Sport, and Molecular/Biology/Genetics journals were often cited by papers published in Medicine/Medical/Clinical journals.

**Table 3:** The top 10 most productive journals for clinical trial research and co-cited journals.

Rank	Journals	Number of publications	Number of citations	Average Number of citations	IF and JCR division(2022)	Co-cited Journals	Number of citations	IF and JCR division (2022)	Centrality
1	Annals of the Rheumatic Diseases	137	15592	115.43	27.4,Q1	Annals of the Rheumatic Diseases	8580	27.4,Q1	0.01
2	Journal of Rheumatology	111	4109	36.15	3.9,Q2	Journal of Rheumatology	4473	3.9,Q2	0.02
3	Rheumatology	64	2321	37.72	5.5,Q1	Arthritis and Rheumatism	3676	—	0.01
4	Clinical and Experimental Rheumatology	61	1102	18.54	3.7,Q2	Arthritis Rheumatology	2503	13.3,Q1	0.01
5	Clinical Rheumatology	49	1158	23.92	3.4,Q2	Rheumatology	1907	5.5,Q1	0.02
6	Arthritis Research Therapy	42	1653	40.77	4.9,Q2	Lancet	1338	168.9,Q1	0.09
7	Arthritis and Rheumatism	33	8284	246.71	—	New England Journal of Medicine	982	158.5,Q1	0.15
8	Rheumatology International	31	988	29.28	4.0,Q2	Arthritis Research Therapy	837	4.9,Q2	0.05
9	Best Practice Research in Clinical Rheumatology	28	745	24.42	5.2,Q1	Clinical and Experimental Rheumatology	783	3.7,Q2	0.09
10	Arthritis Rheumatology	27	1473	54.11	13.3,Q1	Arthritis Care Research	759	4.7,Q2	0.05

### 3.5 Co-cited references and references burst

**Figure 7:**
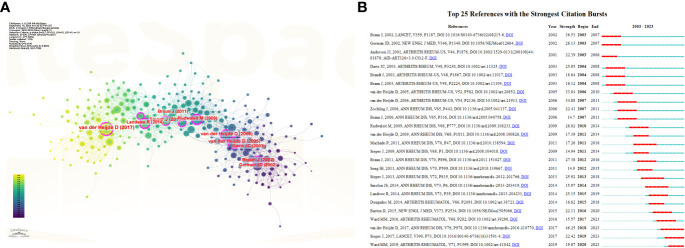
**(A)** Visual analysis of co-cited references in the field of AS clinical trials. **(B)** Cite the top 25 references with the most outbreaks.

**Table 4** summarizes the 10 most frequently cited articles in the field of AS clinical trials. All 10 articles were published between 2003 and 2015, mainly in two journals: Arthritis and Rheumatism and Annals of the Rheumatic Diseases. **Figure 7A** presents an analysis of the co-citations of references using CiteSpace software. The visual co-citation network contained 300 nodes and 1,601 links. This figure shows the first author and release time of the top 10 most-cited references. The 25 references with the strongest citation bursts are also shown in **Figure 7B**. The first reference with the strongest citation explosion (intensity = 36.53) appeared in 2003 and the most recent reference with the strongest citation explosion (intensity = 19.87) appeared in 2020.

**Table 4:** AS clinical trial studies 10 most frequently cited references.

Rank	Title	Year	Journal	Number of Co-citations
1	Secukinumab, an Interleukin-17A Inhibitor, in Ankylosing Spondylitis	2015	New England Journal of Medicine	699
2	Efficacy and safety of infliximab in patients with ankylosing spondylitis - Results of a randomized, placebo-controlled trial (ASSERT)	2005	Arthritis and Rheumatism	644
3	Efficacy and safety of adalimumab in patients with ankylosing spondylitis - Results of a multicenter, randomized, double-blind, placebo-controlled trial	2006	Arthritis and Rheumatism	641
4	Recombinant human tumor necrosis factor receptor, (etanercept) for treating ankylosing spondylitis - A randomized, controlled trial	2003	Arthritis and Rheumatism	599
5	Assessment of outcome in ankylosing spondylitis: an extended radiographic scoring system	2005	Annals of the Rheumatic Diseases	554
6	Ankylosing Spondylitis Disease Activity Score (ASDAS): defining cut-off values for disease activity states and improvement scores	2011	Annals of the Rheumatic Diseases	504
7	Nonsteroidal antiinflammatory drugs reduce radiographic progression in patients with ankylosing spondylitis - A randomized clinical trial	2005	Arthritis and Rheumatism	480
8	A randomised, double-blind, multicentre, parallel-group, prospective study comparing the pharmacokinetics, safety, and efficacy of CT-P13 and innovator infliximab in patients with ankylosing spondylitis: the PLANETAS study	2013	Annals of the Rheumatic Diseases	456
9	Anti-interleukin-17A monoclonal antibody secukinumab in treatment of ankylosing spondylitis: a randomised, double-blind, placebo-controlled trial	2013	LANCET	450
10	Efficacy and Safety of Golimumab in Patients With Ankylosing Spondylitis Results of a Randomized, Double-Blind, Placebo-Controlled, Phase III Trial	2008	Arthritis and Rheumatism	447

**Figure 8:**
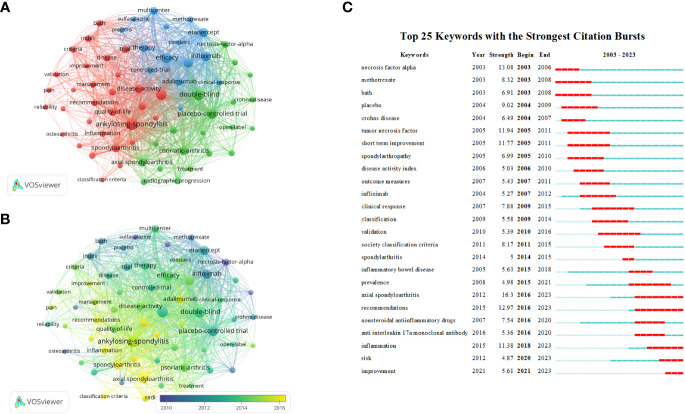
**(A)** Web visualization of the keywords in the AS clinical trials field. **(B)** AS clinical trials in the field of keyword overlay visualization. **(C)** The top 25 references with the most outbreaks cited.

### 3.6 Analysis of the top 10 most-cited articles

By analyzing the most cited articles in the field, the center and focus of research can be identified, thereby assisting beginners in the field to quickly understand the research process and direction. Based on the data available in **Table 4**, the intervention measures, treatment methods, duration, and main outcome indicators of the first 10 highly cited clinical trials were analyzed in detail (**Table 5**). Studies ranged from earlier ones on early nonsteroidal anti-inflammatory drugs and tumor necrosis factor inhibitors to more recent ones on interleukin-17A inhibitors, with more than half of trials using placebo-controlled interventions. The treatment was mostly subcutaneous injection. Almost all of the studies assessed clinical symptom improvement according to the Assessment of Spondyloarthritis International Society 20.

**Table 5:** AS clinical trial study of the top 10 most frequently cited articles treatment measures analysis.

Rank	Title	Intervention Measure	Treatment mode	Duration(W)	Primary outcome measure
1	Secukinumab, an Interleukin-17A Inhibitor, in Ankylosing Spondylitis	Secukinumab vs Placebo	Subcutaneous injection or intravenous loading	52	ASAS20
2	Efficacy and safety of infliximab in patients with ankylosing spondylitis - Results of a randomized, placebo-controlled trial (ASSERT)	Infliximab vs Placebo	Subcutaneous injection	24	ASAS20
3	Efficacy and safety of adalimumab in patients with ankylosing spondylitis - Results of a multicenter, randomized, double-blind, placebo-controlled trial	Adalimumab vs Placebo	Subcutaneous injection	24	ASAS20
4	Recombinant human tumor necrosis factor receptor, (etanercept) for treating ankylosing spondylitis - A randomized, controlled trial	Etanercept vs Placebo	Subcutaneous injection	24	ASAS20
5	Assessment of outcome in ankylosing spondylitis: an extended radiographic scoring system	SASSS vs mSASSS	radiograph	48	1984 Modified New York criteria
6	Ankylosing Spondylitis Disease Activity Score (ASDAS): defining cut-off values for disease activity states and improvement scores	ASDAS	—	24	ASAS
7	Nonsteroidal antiinflammatory drugs reduce radiographic progression in patients with ankylosing spondylitis - A randomized clinical trial	continuous treatment with NSAIDs vs on-demand treatment with NSAIDs	Subcutaneous injection	96	mSASSS
8	A randomised, double-blind, multicentre, parallel-group, prospective study comparing the pharmacokinetics, safety, and efficacy of CT-P13 and innovator infliximab in patients with ankylosing spondylitis: the PLANETAS study	Infliximab (INX) vs CT-P13(a biosimilar to INX)	Subcutaneous injection	30	ASAS20 and ASAS40
9	Anti-interleukin-17A monoclonal antibody secukinumab in treatment of ankylosing spondylitis: a randomised, double-blind, placebo-controlled trial	Secukinumab vs Placebo	Subcutaneous injection	28	ASAS20
10	Efficacy and Safety of Golimumab in Patients With Ankylosing Spondylitis Results of a Randomized, Double-Blind, Placebo-Controlled, Phase III Trial	Golimumab vs Placebo	Subcutaneous injection	24	ASAS20

ASAS, Assessment of Spondyloarthritis International Society; ASAS20,Assessment of Spondyloarthritis International Society 20; ASAS40, Assessment of Spondyloarthritis International Society 40; SASSS, Stoke Ankylosing Spondylitis Spinal Score; mSASSS, modified Stoke Ankylosing Spondylitis Spinal Score; ASDAS, Ankylosing Spondylitis Disease Activity Score; NSAIDs, Nonsteroidal antiinflammatory drugs.

### 3.7 Keywords co-occurrence and burst analysis

**Table 6** summarizes the top 10 keywords occurring most frequently, among which “double blind” and “placebo controlled trial” are keywords related to clinical trials, while excluding keywords related to ankylosing spondylitis clinical trials. The most frequently occurring keywords were “rheumatoid arthritis,” “efficacy,” “infliximab,” “etanercept,” “psoriatia arthritis,” and “therapy.” We used the VOSviewer software to perform keyword co-occurrence analysis, which can identify key points and research frontiers in the field of AS clinical trials. VOSviewer searched for a total of 3,210 keywords, of which 66 appeared more than 30 times, and 65 were screened after combining repeated keywords. A cluster analysis was then performed using the extracted keywords. As shown in **Figure 8A**, three clusters of different colors were obtained. Red represents the largest cluster, which contained 30 keywords associated with “ankylosing spondylitis,” “arthritis,” “bath,” “controlled trial,” “disease activity,” “rheumatoid arthritis,” “inflammation,” “quality of life,” “index,” “pain,” “psoriatic arthritis” and “risk.” The green cluster was the second-largest cluster, containing 20 keywords. These keywords mainly included “active ankylosing spondylitis,” “anti-TNF therapy,” “antitumor necrosis factor,” “crohns disease,” “psoriatic arthritis,” “axial spondyloarthritis,” “nonsteroidal anti-inflammatory drugs,” “radiographic progression” “infliximab,” “methotrexate,” “necrosis factor therapy” and “monoclonal antibody.” Blue was the third largest cluster, with a total of 15 keywords extracted, including “clinical response,” “etanercept,” “multicenter,” “remission,” “efficacy” and “safety.” **Figure 8B** presents a superimposed visualization of keywords, showing the changes that took place in terms of the keywords used over time. Purple nodes indicate early hotspots, while yellow nodes indicate emerging hotspots. The keywords gradually changed from “necrosis factor alpha,” “methotrexate,” “sulfasalazine,” and “tumor necrosis factor” to “inflammation” and “psoriatic,” “arthritis,” “axial spondyloarthritis,” and “remission.” **Figure 8C** shows the results of an analysis performed using the CiteSpace software, showing the top 25 keywords with the strongest reference bursts. The keyword “axial spondyloarthritis” has the strongest explosive power (intensity = 16.3) and is also the keyword with the longest eruption duration, ranging from 2003 to 2006. The earliest keyword for the outbreak was “necrosis factor alpha,” while “inflammation,” “risk” and “improvement” were the latest keywords added in recent years. **Figures 8B, C** that demonstrate that “inflammation,” “psoriatic arthritis” and “axial spondyloarthritis” were the keywords that occurred most frequently recently, indicating that these keywords may represent the current hotspot and future trend regarding clinical trial research on AS.

**Table 6:** Top 10 keywords in terms of frequency regarding AS clinical trials.

Rank	Keyword	Occurrences	Total link strength
1	double blind	373	2294
2	rheumatoid arthritis	345	1859
3	efficacy	292	1853
4	placebo controlled trial	243	1594
5	infliximab	242	1581
6	safety	221	1437
7	etanercept	199	1328
8	psoriatia arthritis	161	927
9	therapy	159	875
10	disease activity	157	893

## 4 Discussion

This study represents the first bibliometric analysis of AS clinical trials that has been implemented to date over the last 20 years. This study mainly used CiteSpace and VOSviewer to analyze 1,212 articles selected from the Web of Science Core Collection. This included the number of global publications focusing on AS clinical trials, the countries or regions where these publications originated, the level of cooperation between institutions and authors, references, and keywords. Together, this enabled us to create a more in-depth analysis of the research hotspots and future development trends in the field of AS clinical trials.

### 4.1 Overview of development in the field of AS clinical trials

Over the past two decades, publications in the field of AS clinical trials have shown a steady upward annual trend (mainly divided into two phases), which began to plateau after a surge in 2005 and 2014, respectively. This may be related to the approval of tumor necrosis factor inhibitors (18) and interleukin-17A inhibitors (19, 20) for clinical use. The number of publications peaked in 2018, when nearly 70% of the articles published in the prior decade were released. This indicated a growing interest in AS among clinical trial practitioners.

From a country or regional perspective, the top three most productive countries in the field of AS clinical trials are the United States, Germany, and the Netherlands, with these three countries also contributing the most to the NC, AC, and H indices. It is worth noting that although China ranks 7th in terms of NP, its NC, AC, and H-index are the lowest among the top 10 most productive countries. This indicates that there is currently an imbalance between the number and quality of clinical trials in the AS field in China. Clinicians in China should pay close attention to current research hotspots, while also placing additional importance on ongoing scientific innovation, and strengthening cooperation with other countries to improve the existing quality of clinical trial articles. Of the 10 most productive institutions, the top five are from Germany, followed by France and the Netherlands. These are also notably the top three countries in terms of AC contribution, which clearly demonstrates that establishing world-class academic institutions is key to solving any existing imbalance in resource development. The presence of such institutions can also improve a country’s scientific research, innovation ability, and academic status.

Of the top 10 most prolific authors, Desiree Van Der Heijde has been the author that has made the most contributions in the field of AS clinical trials over the past 20 years. Van Der Heijde is a member of the Leiden University Medical Center (LUMC), whose team focuses on research in rheumatology, particularly in the treatment of spinal arthritis. Van Der Heijde has led several clinical trials for AS (21–23), and has developed definitions (24), classifications (25), and guidelines for spinal arthritis (26). Van Der Heijde’s team have also validated and improved the classification criteria for axial spinal arthritis (27), helping clinicians to diagnose this disease more accurately. Joachim Sieper and Juergen Braun have been the second most prolific authors after Van Der Heijde; they are both rheumatologists at the Charite Universitatsmedizin Berlin and Rheumazentrum Ruhrgebiet, respectively. Both authors contributed significantly to early AS clinical trials and have collaborated together many times. Collaboration has notably always been an important avenue for scientific research. Sieper and Braun jointly published an article entitled “Ankylosing spondylitis” in the Lancet in 2007 (3); systematically describing the pathogenesis and treatment of AS and pointing out that while tumor necrosis factor blockers are good news for refractory patients, nonsteroidal anti-inflammatory drug therapy and physical therapy remain the preferential long-term treatment options for conventional AS patients. Two recent studies have provided a more detailed description of the structure and function of HLA-B27 (28), suggesting that HLA-B27 may confer evolutionary advantages associated with infection prevention. A joint article published in the New England Journal of Medicine in 2015 reported that a subcutaneous dose of 150mg of secukinumab was shown to significantly improve the signs and symptoms of AS patients after 16 weeks of treatment, indicating a potentially promising direction for subsequent clinical treatment (29).

An analysis of journals and co-cited journals can provide a reliable reference source for researchers’ contributions (30). Of the top 10 most productive journals, Annals of the Rheumatic Diseases and the Journal of Rheumatology are the two most productive specialized journals in the field of rheumatology. Both have published numerous clinical trial articles on the treatment of AS. Annals of the Rheumatic Diseases in particular has the highest NC and AC and is far ahead of other journals in this field. The IF of this journal has also been steadily improving and more valuable research results are expected to be published in the future.

### 4.2 Research hotspots and development trends of AS in clinical trials

The top 10 cited references were published in the top journals of rheumatism and immunity, with most of them having been written by the teams of Van Der Heijde and Sieper (31, 32), a finding which is consistent with the results of our previous studies. As shown in **Table 4**, most articles (33–35) covered in this study centered on clinical trials focused on the effect of tumor necrosis factor inhibitors on AS, with the research time having been concentrated in the early stages. These findings suggest that tumor necrosis factor inhibitors such as adalimumab, infliximab, and golimumab are more effective and better tolerated in the majority of AS patients than traditional rheumatic immune agents such as nonsteroidal anti-inflammatory drugs and glucocorticoids. These clinical trials have laid the foundation for future drug promotion and related research. The majority of the most-cited studies in the past decade have focused on Secukinumab, an interleukin-17A inhibitor (29, 36), which found that this drug rapidly reduced clinical symptoms in patients with AS and can operate as a complementary and alternative treatment for tumor necrosis factor inhibitors.

Interleukin-17 (IL-17) is a pro-inflammatory cytokine derived mainly from TCD4+T cells and helper T subtype 17 cells (37). Studies have found that it is related to matrix destruction, tissue inflammation, and autoimmunity (10, 37). Compared with healthy subjects, the number of TH17 cells in AS patients was significantly increased, and IL-17 in serum and joints were also significantly increased (38). Six IL-17 family members range from A to F. IL-17A, as the first member studied (39), was found to increase the expression of receptor activator of NF-κB ligand in osteoblasts (40), indicating that IL-17A plays an important role in bone remodeling. More studies have found that IL-17A can also increase the expression of adhesion molecules in endothelial cells by stabilizing the chemokines induced by tumor necrosis factor, thus promoting the aggregation of granulocytes to the inflammatory site (41, 42). IL-17, as a potential therapeutic target, plays an important role in immune system diseases. Additionally, no significant adverse reactions and infections have been found in patients since the clinical approval of IL-17A inhibitors. All these findings indicate that the in-depth mining of IL-17 has increasingly become the focus of AS research.

Keywords are the key to retrieving the required articles for a comprehensive analysis. They represent the core and essence of an article, and can best reflect the relevant research hotspots in a specific field. This study also analyzed the evolution of keywords in the field of AS clinical trials over time through superposition visualization, which, to a certain extent, can better reflect changes in research hotspots, thus guiding the future direction of this research. Among the top 10 most-cited keywords, “double-blind” and “placebo-controlled trial” are the most commonly used methods in clinical trials in this field; this result is also consistent with the analysis of interventions in the top 10 most-cited articles displayed in **Table 5**. In clinical trials, placebos not only have no therapeutic effect on the observed disease, but can also simulate the possible adverse effects of the experimental drug (43). Therefore, it is very important to establish a placebo group in controlled clinical trials (44), a format which is increasingly being chosen by most clinical workers. Rheumatoid and psoriatic arthritis are the two diseases most closely related to AS, and their treatment methods are similar. “Infliximab” and “etanercept,” two tumor necrosis factor inhibitors (45, 46), are mostly used in clinical trials and have been shown to be both effective and well tolerated. According to the combined keyword cluster, “efficacy” and “safety” are keywords closely related to RCT, which indicates that the development of evidence-based medicine for AS is becoming more and more mature (47). As can be seen from the superimposed visual analysis, research in AS clinical trials has shifted from focusing on drug effectiveness to focusing on drug tolerance. Combined with the keyword outbreak analysis, “inflammation,” “risk” and “improvement” have been the emerging keywords in recent years. This indicates that clinicians are now more focused on the long-term quality of life of patients (48–50). Through the superimposed visual analysis of keywords, combined with the analysis of highly cited articles displayed in **Table 4** and **Table 5** in the past 10 years, it can be predicted that “placebo-controlled trial,” “double-blind,” “infliximab,” “improvement,” and other keywords may still break out in the future. However, “Interleukin-17” and “Secukinumab” will be the keywords for future outbreaks in this field. Based on the above analysis of various keywords, it can be seen that future research hotspots in the field of AS clinical trials will continue focus on the development of immunotherapy drugs for the treatment of this disease. This is especially the case with regard to randomized controlled trials studying the effects of tumor necrosis factor inhibitors and interleukin inhibitors. However, researchers will also continue to pay more attention to the long-term effectiveness of drugs in improving the clinical symptoms of patients and reducing the recurrence rate of AS, to enable patients to continue to pursue a higher quality of life.

## 5 Limitations

It is important to note that this study possesses some limitations. Although Web of Science is one of the most authoritative literature search tools, it does not cover all of the literature related to this study. In addition, we only searched for articles written in English and ignored those published in other languages, which may have led to a bias in the results.

## 6 Conclusion

To the best of our knowledge, this study represents the first bibliometric analysis in the field of AS clinical trials. It identifies current research hotspots and directions in this field, and aims to predict future research developments, which may provide valuable information for future clinicians. Future studies should focus on the continued exploration of tumor necrosis factor and interleukin inhibitors and the conducting of placebo-controlled trials.

## Data availability statement

The original contributions presented in the study are included in the article/supplementary material. Further inquiries can be directed to the corresponding authors.

## Author contributions

WZ: Writing – original draft. ML: Data curation, Formal analysis, Writing – original draft. XL: Writing – original draft. XW: Data curation, Formal analysis, Writing – original draft. YL: Supervision, Writing – review & editing. JY: Supervision, Writing – review & editing.
